# Traumatic posterior Atlanto-axial dislocation: case report of an atypical C1-C2 dislocation with an anterior arch fracture of C1

**DOI:** 10.1186/s12891-019-3005-2

**Published:** 2019-12-20

**Authors:** Soufiane Ghailane, Mohammad A. Alsofyani, Vincent Pointillart, Houssam Bouloussa, Olivier Gille

**Affiliations:** 1 Spine Surgery Unit 1, C.H.U Tripode Pellegrin, Université de Bordeaux, Place Amélie Raba Léon, 33076 Bordeaux, France; 2 Department of Orthopedic Surgery, College of Medicine, University of Hail, P O Box, 2440 Hail, Kingdom of Saudi Arabia; 3 Division of Pediatric Orthopaedic Surgery, Children’s Hospital of Pittsburgh of UPMC, 4401 Penn Ave, Pittsburgh, PA 15224 USA

**Keywords:** Cervical spine dislocation, Posterior Atlantoaxial dislocation, Closed manual reduction

## Abstract

**Background:**

An atypical case of a traumatic posterior C1-C2 dislocation with an anterior arch fracture of C1 is reported. A novel conservative treatment for this rare lesion is described.

**Case presentation:**

An eighty-nine-year-old male fell off a ladder at home and presented with an acute traumatic cervical spine trauma, which we believe involved a distraction mechanism. The patient was neurologically intact; he denied any weakness, numbness or paresthesia. A preoperative CT-scan demonstrated a posterior dislocation with an anterior arch of C1 fracture. Conservative management was elected. Reduction was achieved by closed manual reduction under general anesthesia. A postoperative CT demonstrated a complete reduction of the atlanto-axial dislocation.

**Conclusion:**

Based on this case report and relevant literature, we present an unusual lesion of the upper cervical spine treated nonoperatively with closed manual reduction under general anesthesia. To date, there is no available consensus for the management of these lesions.

## Background

The combination of posterior atlantoaxial joint dislocation and anterior arch fracture of C1 is uncommon: an epidemiological review of 116 cases of upper cervical spine injuries does not report it [1]. The vast majority of traumatic C1-C2 dislocations are anterior [2]. We present an atypical case of a traumatic posterior C1-C2 dislocation with an anterior arch fracture of C1 treated by a novel approach.

## Case presentation

**Fig. 1:**
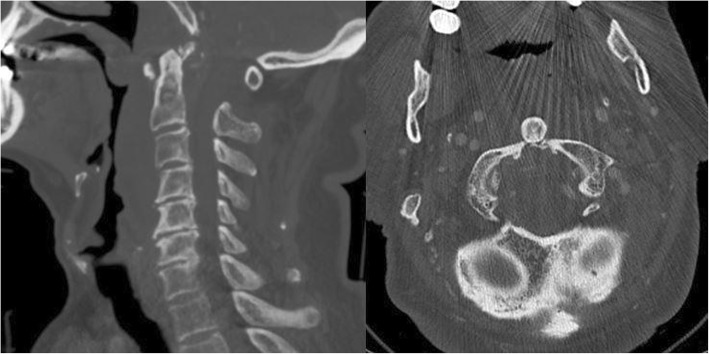
Pre-reduction mid-sagittal and axial CT slices of the cervical spine

**Fig. 2:**
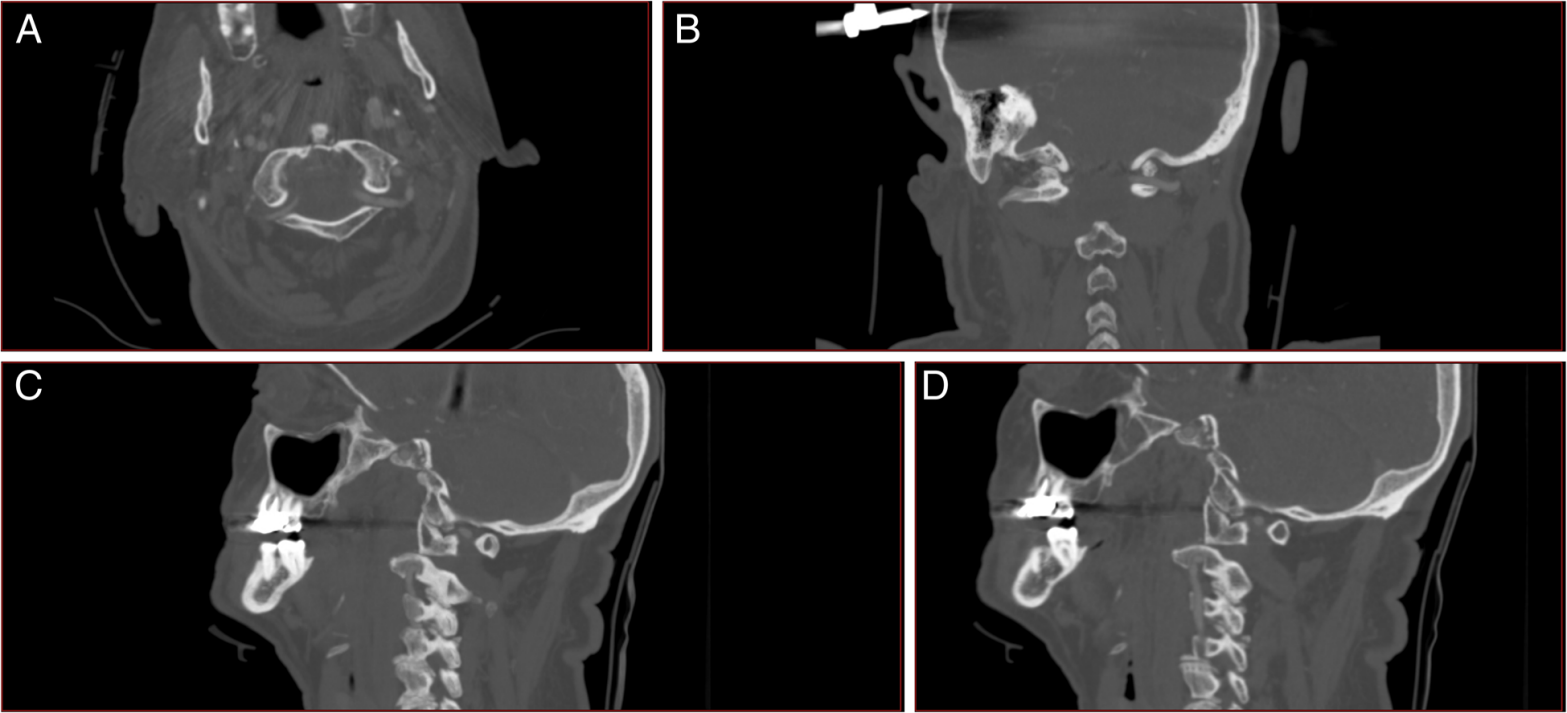
CT-angiography of the cervical spine showing maintained perfusion of the vertebral arteries. No signs of thrombosis or dissection. The left vertebral artery is dominant. **a**. Axial slice through the lateral masses of C1. **b**. Coronal slice through the 3rd segment of the vertebral arteries. **c**. Left-sided parasagittal slice through the dislocated C1-C2 joint. **d**). Right-sided parasagittal slice through the dislocated C1-C2 joint

An 89-year-old male, with a history of stable angina, was admitted to our unit for blunt head and neck trauma with no loss of consciousness after falling off a ladder at home. Upon arrival at the emergency department, he complained of upper cervical midline pain. He denied any subjective neurological signs such as weakness, numbness or paresthesia. On examination, the patient was awake and alert, the Glasgow Coma Scale (GSC) was 15 and vital signs were stable. Midline tenderness at the C1 and C2 levels was triggered by palpation. The rest of the neurological exam, including the rectal exam, was strictly normal and no signs of myelopathy or radiculopathy were found. Cervical spine X-rays and computed tomography (CT) slices demonstrated a posterior dislocation of the atlantoaxial joints with an anterior arch fracture of C1 (Fig. 1). There was no odontoid process fracture. A preoperative CT-angiography ruled out any vertebral artery injury (Fig. 2). There was no injury to the internal carotid arteries either. The patient case was discussed preoperatively during our local trauma round for decision-making.

### Treatment

**Fig. 3:**
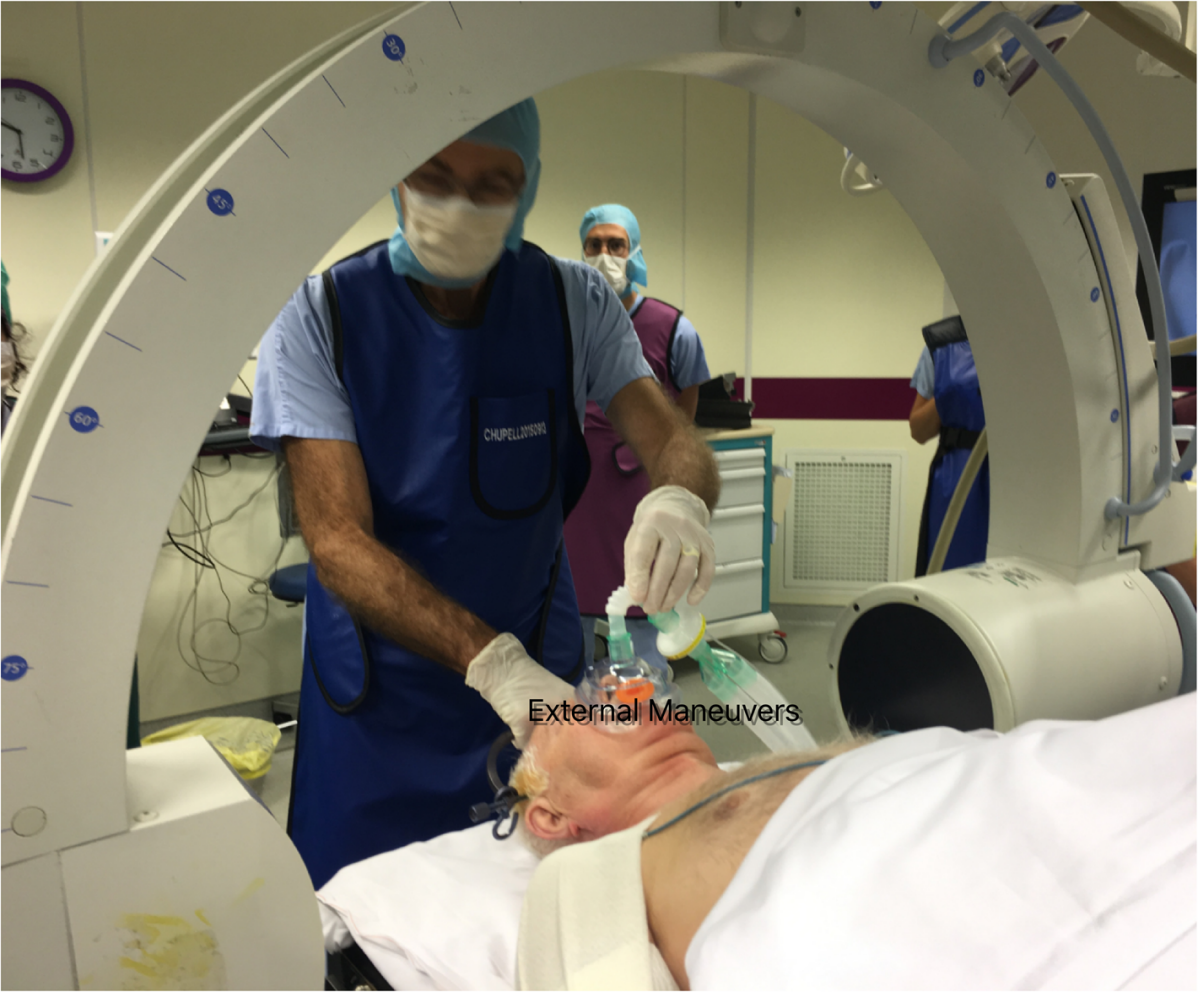
External maneuvers under fluoroscopy

**Fig. 4:**
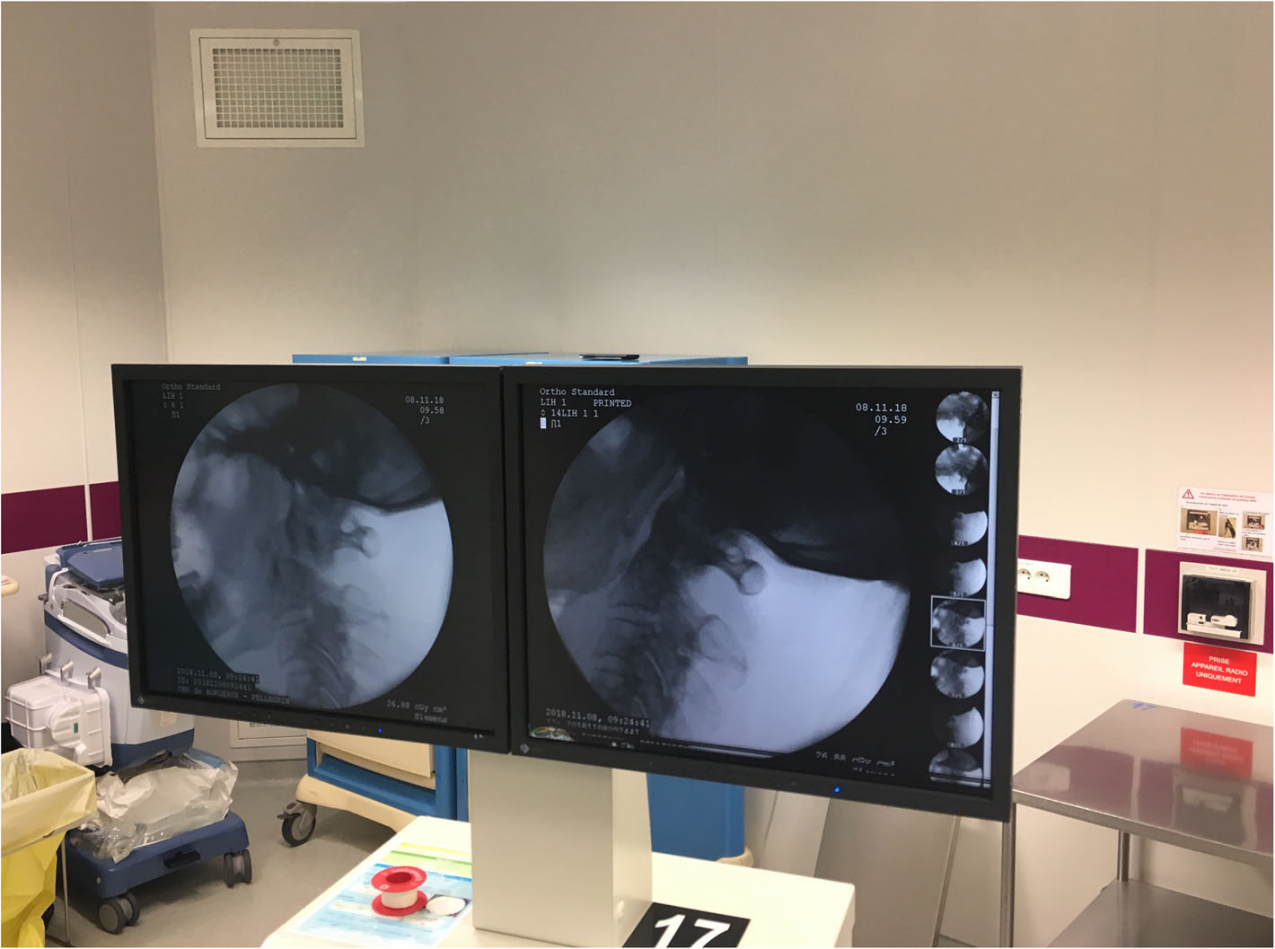
Intraoperative fluoroscopic images before and after reduction

**Fig. 5:**
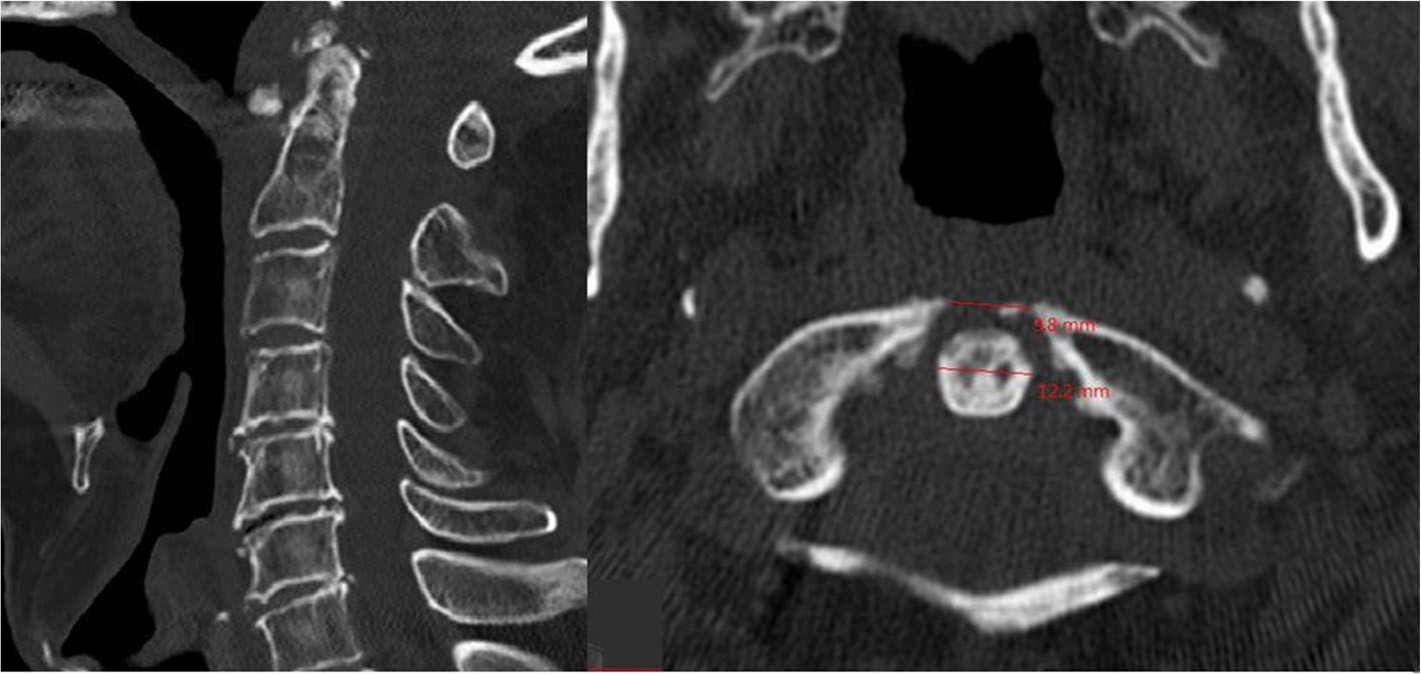
Postoperative mid-sagittal and axial CT slices showing a normalization of the C1-C2 alignment and ADI

**Fig. 6:**
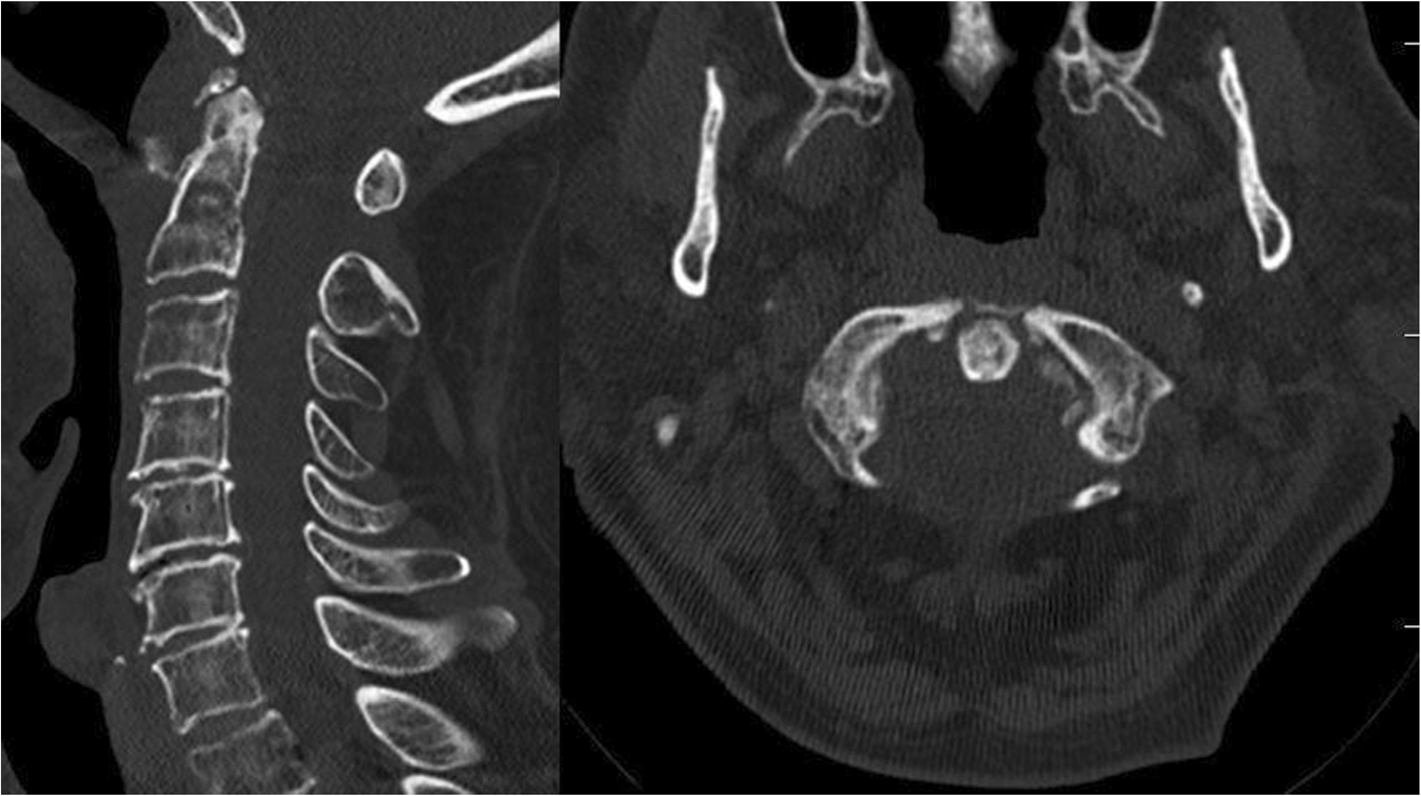
2-month follow-up mid-sagittal and axial CT slices demonstrating a maintained reduction of the anterior arch of C1

**Fig. 7:**
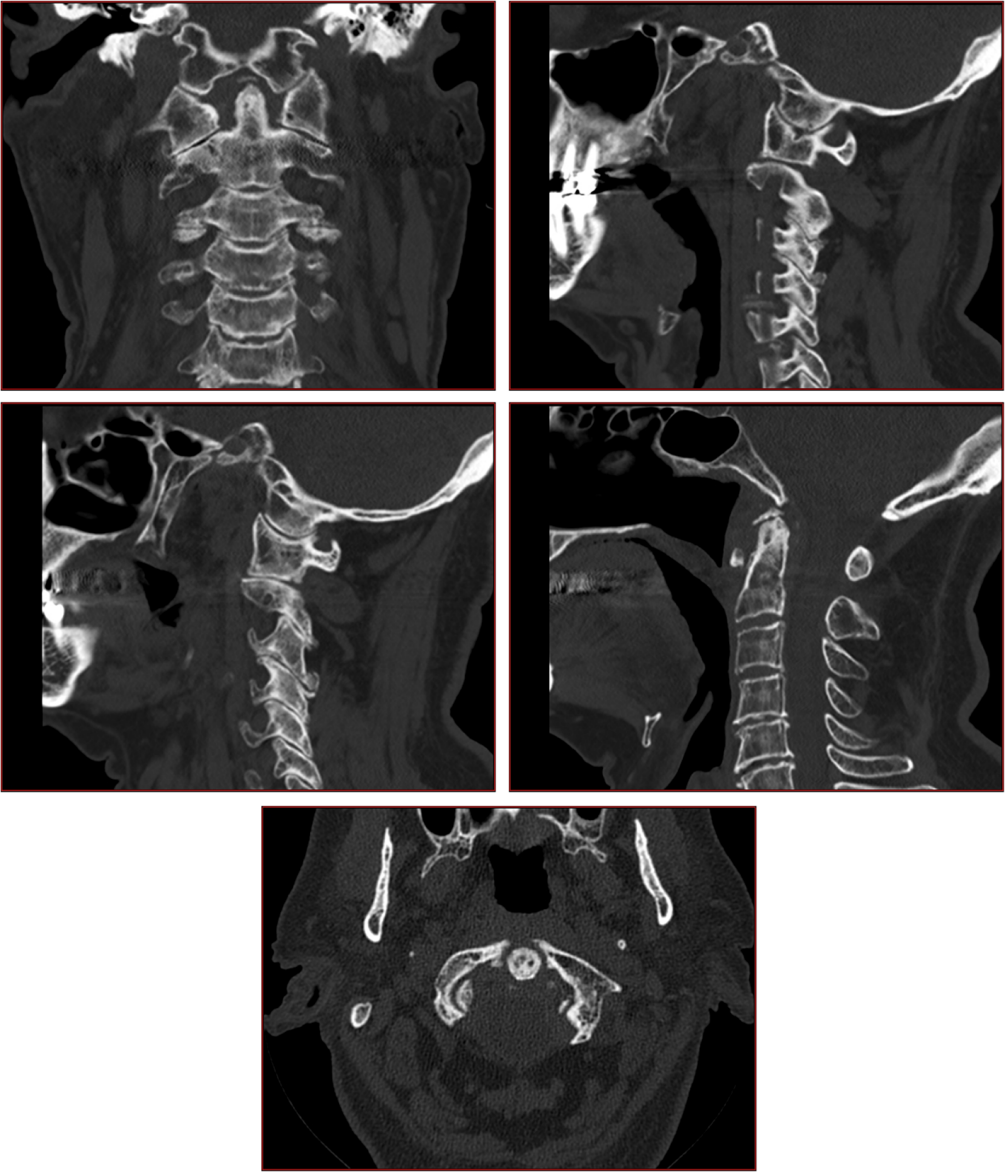
8-month follow-up CT slices demonstrating a maintained reduction of the anterior arch of C1 and subtle signs of healing on the left side of the fracture of the anterior arch of C1. **a**. Coronal slice through the dens of C2. **b**. Right-sided parasagittal slice through the C1-C2 joint. **c**. Left-sided parasagittal slice through the C1-C2 joint. **d**. Mid-sagittal slice displaying a normalized ADI. **e**). Axial slice through the tip of C2

Under general anesthesia, the patient was placed in the prone position with C-arm imaging monitoring. External maneuvers were applied to achieve a satisfactory closed manual reduction of the atlantoaxial dislocation (Fig. 3). A first operator applied moderate traction in hyperextension with a Mayfield clamp while a second operator manually pushed the odontoid trans-orally first in hyperextension and then flexion of the neck under fluoroscopy (Fig. 4). Postoperative cervical spine X-rays and CT slices revealed a complete reduction of the C1-C2 dislocation (Fig. 5). The patient was immobilized with a rigid cervical collar for 2 months. The treatment was well tolerated by the patient. He did not present any skin ulcerations in the interval. At a two-month follow-up, the clinical outcome was favorable (Visual Analog Scale (VAS) of the cervical spine 3/10). The patient presented no major functional complaint. The cervical spine X-rays and CT slices revealed a complete reduction of the atlantoaxial joints (Fig. 6). Dynamic X-rays demonstrated a satisfactory range of motion in flexion and extension (Flexion 50° and Extension 60°). At last follow-up (8 months), the patient was completely asymptomatic. He did not complain of any neck pain. A CT-scan showed a maintained reduction and a subtle bony healing of the anterior arch of C1 (left side) (Fig. 7).

## Discussion and conclusion

**Fig. 8:**
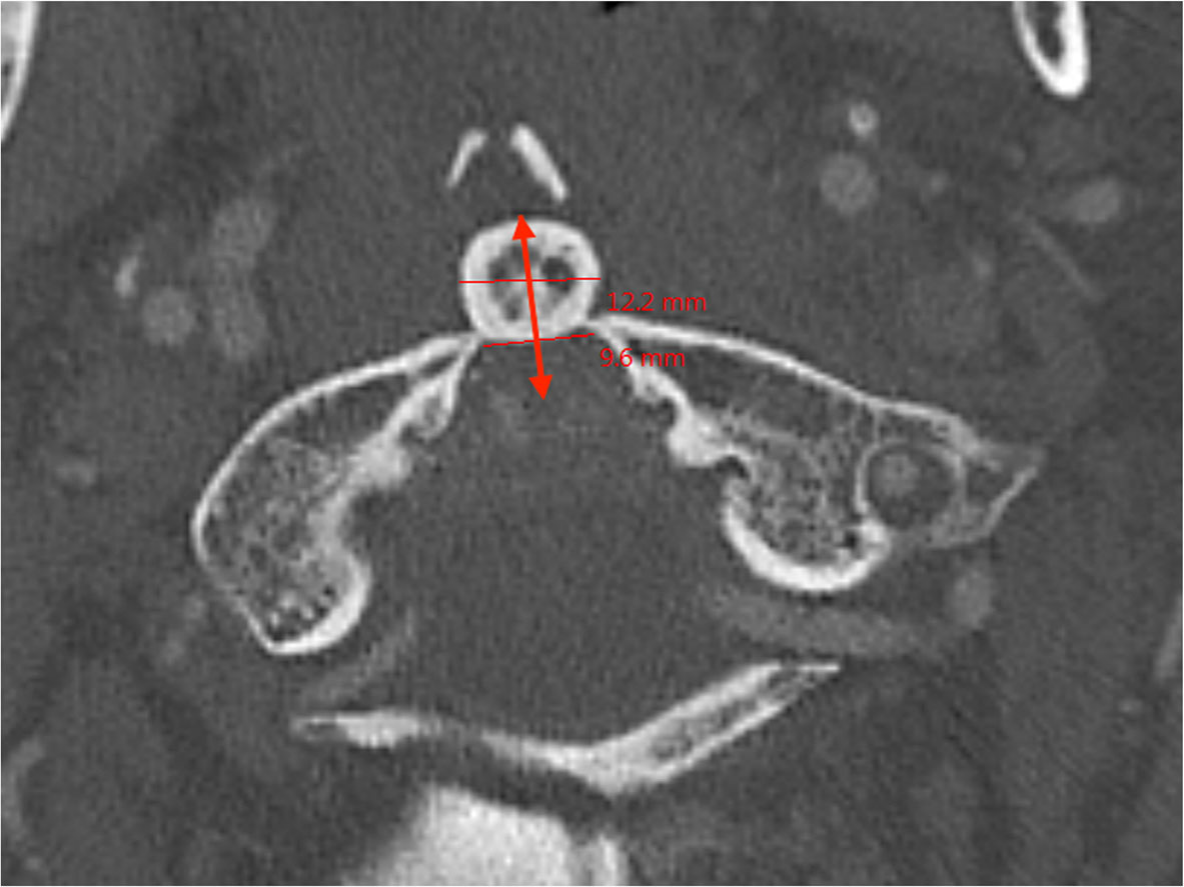
Axial slice of pre-reduction CT showing that the distance between the two fragments of the anterior arch of C1 less than the diameter of the odontoid process. The arrow displays the negative ADI from the anterior aspect of the dens to the posterior aspect of the anterior arch of C1

A review of the English literature found a near-identical case. Yet, it was a posterolateral dislocation managed by closed reduction and Magerl C1–C2 transarticular screw fixation coupled with a modified Brooks fusion [3]. The clinical stability of the atlantoaxial joints depends on both osseous and ligamentous structures. These lesions must have some degree of associated ligamentous injury or rupture (i.e transverse, alar, and/or capsular ligaments). Therefore, the current scientific literature recommends closed reduction followed by C1–C2 arthrodesis as a gold-standard treatment. To our knowledge, conservative management of a posterior C1-C2 dislocation with an anterior arch fracture of C1 has not yet been reported. The mechanism of posterior atlantoaxial dislocation can differ, often resulting in a high-grade hyperextension mechanism [4]. The anterior arch fracture may be secondary to the support of the dens on the arch with shear forces leading to a vertical fracture. In this case, the distance between the two fragments of the anterior arch of C1 is less than the diameter of the odontoid process (Fig. 8). These findings suggest that the odontoid process passed just below the anterior arch of C1.

Vertebral artery compromise following cervical injuries is a known complication [5]. Because of their high-energy mechanism, C1–C2 dislocations are always life-threatening conditions. Therefore, a systematic trauma assessment should always be performed before definitive management of the cervical injury. In this case, preoperative CT-angiography ruled out any vertebral artery injury.

The stability of the upper cervical spine differs from that of the atlantoaxial joints. C1 does not have a vertebral body; it is instead formed by a posterior and anterior arch that surrounds the spinal cord, which is posterior to the dens. All these factors constitute an entire joint system mostly reliant on anterior and posterior atlantoaxial ligaments and the transverse ligament of the atlas [6, 7].

Traumatic C1–C2 antero-posterior dislocations associated with a fracture of the anterior arch of C1 are unusual lesions. According to the literature, the most common fracture associated with atlantoaxial dislocations is the odontoid fracture [8].

In 1986, Autrique et al. [9] described two cases of atlantoaxial dislocation associated with odontoid fracture. One case was treated with halo-vest traction for 6 months while the other was managed by occiput to C3 fusion.

Until the beginning of this century, C1-C2 dislocations with associated odontoid fractures had been described by 11 authors [8, 10–19] . Two cases were managed by halo-vest immobilization for 3–4 months [18, 19], another case was treated by anterior C1-C2 fixation according to Vaccaro’s technique [13], and all other cases were treated by C1-C2 posterior fixation [8, 10–12, 14–17]. One important pitfall of closed reduction techniques is overdistraction [20].

In this case, the authors’ choice was based on the analysis of the injury mechanism. The authors believed that there was no ligamentous instability due to the posterior nature of the dislocation. Most dislocations are anterior and they are responsible for dangerous instability due to a traumatic rupture of the transverse ligament [2]. These dislocations cause an atlantodental interval (ADI) increase. This could have been documented by an MRI, but a clinical decision was made at that time not to perform a preoperative MRI, in order to avoid any treatment delay. Furthermore, the ADI was significantly changed. Yet, the ADI was negative, which is an exceptional finding. In the case of a negative ADI, the dens of C2 migrates anteriorly as was shown in the preoperative CT (Fig. 8) and not posteriorly against the spinal cord. For these reasons, the transverse ligament was deemed intact, which would preclude any anterior instability. Anterior instability by dislocation typically ruptures the transverse ligament and increases the ADI. Regarding the apical ligament, the alar ligaments and the longitudinal band, we believed they were not necessarily ruptured though an MRI would have been necessary to prove it.

Nonsurgical management is not adapted under all circumstances. For this specific case, we believe that the anterior arch of C1 may possibly heal and that the transverse ligament was not ruptured due to the mechanism of injury. However, this cannot be generalized and we certainly do not recommend against performing preoperative MRIs in this scenario as long as it does not delay treatment. Nonsurgical management covers the use of a soft collar, a rigid collar or a halo-vest immobilization. In this case, the choice of a rigid collar was motivated by the age of the patient (89 years old). For patients older than 88 years old with C2 fractures, data from the Swedish Patient Registry and the Swedish Cause of Death Registry show that non-surgical treatment should be primarily attempted [21]. Though it would have been mechanically “ideal” to maintain the reduction with halo-vest immobilization, for increased rotational control, this option was thought to be more dangerous. Indeed, our experience, which is supported by the current literature, is that older patients poorly tolerate halo-vest immobilization (confusion, delirium, increased risk of falls, increased mortality risk) [22–24]. Moreover, because the mechanism of the injury was not rotational, we felt that the best compromise of stability versus safety was to elect a rigid collar. Yet, we acknowledge the risks of this treatment plan: less rotational control with increased risk of secondary instability or fracture displacement, risk of maceration, irritation, allergy, skin ulcers [25]. The management of this case cannot be generalized to younger populations with a higher activity status.

Posterior atlantoaxial dislocations with an isolated fracture of the anterior arch of C1 (without an odontoid process fracture) represent an extremely rare injury that may be safely managed by closed manual reduction under C-arm guidance and rigid immobilization.

Major complications include iatrogenic vertebral artery injury, iatrogenic cervical spine instability, and life-threatening distraction lesions due to skull traction by halo-frame. We believe that being mindful of all these potential complications may enable surgeons to safely perform a closed manual reduction of this lesion using fluoroscopy in the setting of conservative treatment.

## Data Availability

Data sharing is not applicable to this article as no datasets were generated or analysed during the current study.

## Abbreviations

ADI: Atlantodental interval
CT: Computed tomography
VAS: Visual analog scale
